# Distinguishing blood and lymph vessel invasion in breast cancer: a prospective immunohistochemical study

**DOI:** 10.1038/sj.bjc.6603152

**Published:** 2006-05-02

**Authors:** G G Van den Eynden, I Van der Auwera, S J Van Laere, C G Colpaert, P van Dam, L Y Dirix, P B Vermeulen, E A Van Marck

**Affiliations:** 1 Translational Cancer Research Group, Lab Pathology University of Antwerp/University Hospital Antwerp, Antwerp, Belgium; 2 Translational Cancer Research Group, Oncology Center, General Hospital St-Augustinus, Oosterveldlaan 24, B-2610 Wilrijk, Belgium

**Keywords:** blood vessel invasion, lymph vessel invasion, breast cancer, D2-40

## Abstract

Recently, peritumoural (lympho)vascular invasion, assessed on haematoxylin–eosin (HE)-stained slides, was added to the St Gallen criteria for adjuvant treatment of patients with operable breast cancer (BC). New lymphatic endothelium-specific markers, such as D2-40, make it possible to distinguish between blood (BVI) and lymph vessel invasion (LVI). The aim of this prospective study was to quantify and compare BVI and LVI in a consecutive series of patients with BC. Three consecutive sections of all formalin-fixed paraffin-embedded tissue blocks of 95 BC resection specimens were (immuno)histochemically stained in a fixed order: HE, anti-CD34 (pan-endothelium) and anti-D2-40 (lymphatic endothelium) antibodies. All vessels with vascular invasion were marked and relocated on the corresponding slides. Vascular invasion was assigned LVI (CD34⊕ or ⊖/D2-40⊕) or BVI (CD34⊕/D2-40⊖) and intra- (contact with tumour cells or desmoplastic stroma) or peritumoural. The number of vessels with LVI and BVI as well as the number of tumour cells per embolus were counted. Results were correlated with clinico-pathological variables. Sixty-six (69.5%) and 36 (37.9%) patients had, respectively, LVI and BVI. The presence of ‘vascular’ invasion was missed on HE in 20% (peritumourally) and 65% (intratumourally) of cases. Although LVI and BVI were associated intratumourally (*P*=0.02), only peritumoural LVI, and not BVI, was associated with the presence of lymph node (LN) metastases (*p*_peri_=0.002). In multivariate analysis, peritumoural LVI was the only independent determinant of LN metastases. Furthermore, the number of vessels with LVI was larger than the number of vessels with BVI (*P*=0.001) and lymphatic emboli were larger than blood vessel emboli (*P*=0.004). We demonstrate that it is possible to distinguish between BVI and LVI in BC specimens using specific lymphatic endothelium markers. This is important to study the contribution of both processes to BC metastasis. Furthermore, immunohistochemical detection of lymphovascular invasion might be of value in clinical practice.

Breast cancer (BC) is the most frequent cause of death in women between 35 and 55 years of age (Uzzan *et al*, 2004). Loco-regional spread and recurrence of the disease can be debilitating, but metastasis to distant organs is the leading cause of breast cancer (BC)-related death. One of the very early steps in the metastatic cascade is (lympho)vascular invasion, or the penetration of tumour cells into lymph and/or blood vessels in and around the primary tumour. Therefore, tumour cell emboli in lymph and blood vessels are considered to be the morphological correlates of BC metastasising to loco-regional lymph nodes (LNs) and distant haematogenous sites, respectively. The presence of lymphovascular invasion indeed has been correlated to the presence of LN metastases and to poor prognosis in patients with BC (Sampat *et al*, 1977; Nealon *et al*, 1979, 1981; Dawson *et al*, 1982, 1986; Weigand *et al*, 1982; Bettelheim *et al*, 1984; Berger *et al*, 1988; Rosen *et al*, 1989, 1991; Lee *et al*, 1990; Clayton, 1991; Clemente *et al*, 1992; Neville *et al*, 1992; Fisher *et al*, 1993a, 1993b; Lauria *et al*, 1995). Recently, interest in (lympho)vascular invasion has been increased owing to addition of peritumoural (lympho)vascular invasion to the St Gallen criteria for selection of adjuvant systemic treatment in operable BC (Goldhirsch *et al*, 2005). In these criteria, lymphovascular invasion is detected on haematoxylin–eosin (HE) sections that do not allow distinction between blood vessel invasion (BVI) and lymph vessel invasion (LVI).

Historically, different methodological problems have hampered visualisation of LVI and BVI in resection specimens of patients with BC. One of the major challenges has been to distinguish intratumoural lymph and blood vessels on HE slides from retraction artefacts caused by tissue fixation and processing. Most authors only included vessels with a clear-cut endothelium (Sampat *et al*, 1977; Nealon *et al*, 1979, 1981; Dawson *et al*, 1982, 1986; Weigand *et al*, 1982; Bettelheim *et al*, 1984; Berger *et al*, 1988; Rosen *et al*, 1989, 1991; Orbo *et al*, 1990; Clayton, 1991; Clemente *et al*, 1992; Neville *et al*, 1992; Fisher *et al*, 1993a, 1993b; Pinder *et al*, 1994; Lauria *et al*, 1995), missing small and collapsed intratumoural vessels or vessels completely filled with tumour cells. Therefore, some authors only studied peritumoural LVI and BVI (Clemente *et al*, 1992; Neville *et al*, 1992; Lauria *et al*, 1995) or used morphological and topographical criteria to identify LVI (Orbo *et al*, 1990).

Another methodological problem has been distinguishing between blood and lymph vessels. Until recently, the lack of lymph vessel-specific markers made it very difficult to specifically study BVI or LVI. Therefore, some authors investigated ‘vascular invasion’, including both LVI and BVI (Berger *et al*, 1988; Pinder *et al*, 1994). Others tried to distinguish between both using blood vessel-specific characteristics such as typical blood vessel morphology or the presence of red blood cells or fibrin cloths (Weigand *et al*, 1982; Lauria *et al*, 1995). Lee *et al* (1986, 1990) used morphologic and immunohistochemical criteria and more recently, blood vessels were identified based on FVIII-antigen immunohistochemical or van Gieson elastica stains (Kato *et al*, 2002, 2003).

During the last decade, several specific markers for lymphatic endothelium have been discovered, such as Prox-1, a transcription factor, Lyve-1, a hyaluronan receptor, podoplanin, a glomerular podocyte membrane protein and D2-40. Recently, it has been shown that the D2-40 antibody specifically recognises podoplanin (Schacht *et al*, 2005) and that D2-40 and podoplanin are the most sensitive and specific antibodies for the detection of lymphatic endothelium (Evangelou *et al*, 2005). In BC, we previously demonstrated that D2-40 is the best marker for lymphatic endothelium (Van der Auwera *et al*, 2005). Used in combination with panendothelial markers such as CD34 or CD31, it is now possible to differentiate between BVI and LVI and to study the role of both processes in BC metastasis. Recent data (reviewed by Pantel *et al*) have supported BC metastasis models hypothesising that lymphatic and haematogenous dissemination in BC are two complementary and specific pathways (Pantel and Brakenhoff, 2004). Therefore, the aim of this study was to evaluate a technique using a combined immunohistochemical expression profile to differentiate on an individual vessel basis between LVI and BVI in a consecutive series of patients with BC. Furthermore, we obtained quantitative data on LVI and BVI in BC and correlated the presence of LVI and BVI to other clinico-pathological variables.

## MATERIALS AND METHODS

### Patient selection

From 18 March 2005 until 19 August 2005, all tumourectomy, quadrantectomy or (modified) mastectomy specimens containing invasive BC examined at the Department of Pathology, General Hospital AZ St-Augustinus, Wilrijk, Belgium, were prospectively included in this study after written informed consent. As the investigators had no access to clinico-pathological data, all 109 included resection specimens were processed and analysed as described below. When the anonymisation code was broken, one patient with documented distant metastases and five patients with a local recurrence were excluded. From eight patients with more than one resection specimen, only the first was taken into account. Therefore, 95 patients with operable breast carcinoma were included in this study. One patient refused to have an axillary lymphadenectomy, hence no information on LN status was available in this patient. Table 1 summarises the clinico-pathological data. Age, tumoural (T) and nodal (N) status, histological type, tumour grade, oestrogen (ER) and progesterone receptor (PR) status, p53 and HER2/neu oncoprotein status were recorded by review of the pathology files. Tumours were histologically graded according to the Nottingham modification of the Bloom and Richardson histological grading scheme (Bloom and Richardson, 1957; Elston, 1987). Tumoural and N status were assigned according to the tumour node metastasis classification of the American Joint Committee on Cancer (Green *et al*, 2002). Lymph nodes were examined according to the standard pathology procedure in our institution: HE step sections every 200 *μ*m for sentinel LNs and one HE section every 3 mm for non-sentinel LNs. No immunohistochemical techniques were used for detection of tumour cells in LNs. Furthermore, the presence and size of a fibrotic focus and the growth pattern of the tumour were assessed on the HE slides, as defined previously (Hasebe *et al*, 1998; Colpaert *et al*, 2001, 2003b). A fibrotic focus is defined as a scar-like area, consisting of fibroblasts and collagen fibres, that occupies various percentages of the centre of an invasive ductal carcinoma of the breast. In the infiltrative growth pattern, carcinoma cells infiltrate between pre-existing breast parenchymal structures, without significant disturbance of the breast architecture. In the expansive growth pattern, the tumour forms a well-circumscribed nodule consisting of carcinoma cells and desmoplastic connective tissue. Pre-existing breast parenchymal structures are not present inside the tumour but are pushed aside by the expansively growing nodule. The growth pattern is mixed infiltrative-expansive, when the tumour consists of a central expansive nodule surrounded by carcinoma cells showing an infiltrative growth pattern.

### (Immuno)histochemistry

After routine histopathological examination, three consecutive 4 *μ*m sections of all formalin-fixed paraffin-embedded (FFPE) tissue blocks containing invasive carcinoma were cut and numbered. These slides were (immuno)histochemically stained in a fixed order: the first section of each block was stained with HE, the second with antibodies against CD34 (panendothelium marker) and the third with antibodies against D2-40 (lymphatic endothelium marker). The CD34 and D2-40 immunohistochemistry (IHC) were performed on the Dako Autostainer (Dako, Glostrüp, Denmark) using the Envision Dual Link + as the detection system (Dako). The CD34 (dilution 1/50, Clone Qbend10, Dako) and D2-40 (dilution 1/100, Dako) primary antibodies were both incubated for 30 min.

### Assessment of blood and lymph vessel invasion

**Figure 1:**
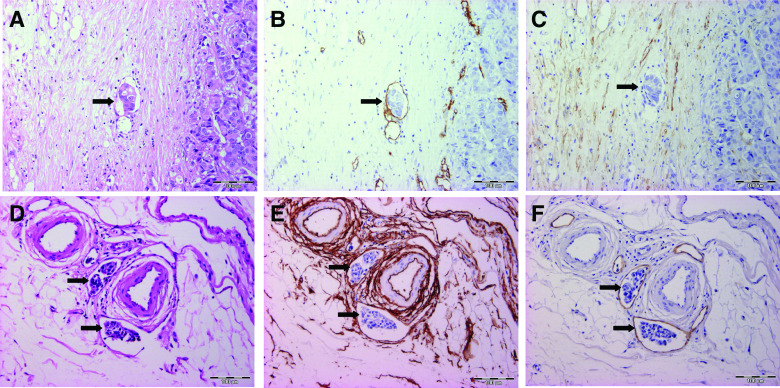
Overview of the histological and immunohistochemical stainings on consecutive slides, used to differentiate between BVI (upper row: **A**, **B** and **C**) and LVI (lower row: **D**, **E** and **F**). Tumour cell emboli are indicated with black arrows. **A** and **D**: HE staining showing the presence of vascular invasion. **B** and **E**: On CD34 staining, both blood (**C**) and lymph (**F**) vessel endothelium stain positive. Furthermore, normal breast stromal cells are also CD34 positive (**E**). **E** and **F**: On D2-40 staining, the endothelium of vessels with BVI (**B**) and LVI (**E**) are respectively negative and positive. Desmoplastic stromal cells are also D2-40 positive (**C**). (BVI=blood vessel invasion, LVI=lymph vessel invasion).

First, all slides (HE, CD34 and D2-40) were screened for (lympho)vascular invasion using strict criteria (*HE*: tumour cells within an endothelium-lined vessel-like structure; *CD34 or D2-40*: tumour cells within an immunohistochemically positive vessel-like structure). Every slide was assessed by two investigators (GVdE and CC) without access to the data of other sections of the same resection specimen or other stainings of the same section. Every vessel with tumour cell invasion according to the criteria on one of the three consecutive sections was relocated on the other slides and assigned BVI or LVI based on the immunohistochemical staining profile (Figure 1). These blood and lymph vessels were also tracked on the corresponding HE stain and were scored as ‘picked-up on HE’ if the vascular invasion had also been marked on HE or ‘missed on HE’ if the vascular invasion had not been seen on HE. Tumour cell invasion in a vascular structure marked on the HE stain that was neither D2-40 nor CD34 positive was called ‘overdiagnosis on HE’. Table 2 schematically shows the different possibilities. Furthermore, the foci of BVI and LVI were scored as intra- (in contact with tumour cells or desmoplastic stroma) or peritumoural and the size of every focus of BVI or LVI was assessed by counting the number of intravascular tumour cells.

### Statistical analysis

Statistical analysis was performed with the SPSS 12.0 software package. A *P*-value⩽0.05 was considered statistically significant, a 0.5<*P*-value⩽0.1 was considered a trend towards statistical significance. Normality was tested with a Kolmogorov–Smirnov test assuming normality of data if *P*⩾0.2. As continuous data (e.g. number of vessels with LVI or BVI, size of lymphatic and blood vessel emboli) were not normally distributed, the median (25–75th percentile) value is reported and compared between different groups with a Mann–Whitney *U*-test. For analysing associations between categorical variables (e.g. presence of LVI or BVI, presence of LN metastases, ER status and PR status), the *χ*^2^ test or – when the assumptions of the *χ*^2^ test were not met – the Fisher's exact test, was used. To build a multivariate model predicting axillary LN involvement, a logistic regression with backward procedure including intra- and peritumoural ‘vascular’ invasion, LVI and BVI was performed.

## RESULTS

### (Lympho)vascular invasion *vs* number of blocks

A total of 3297 vessels (661 intra and 2636 peritumoural) with LVI and 135 vessels with BVI (76 intra and 59 peritumoural) were demonstrated in consecutive sections of 446 FFPE tissue blocks. The median number of blocks per patient was four (range 1–20). The number of FFPE blocks investigated was significantly correlated with the size of the tumour (*ρ*=0.40, *P*<0.001). Lymph vessel invasion (*P*=0.01) and BVI (*P*<0.001) were more frequently demonstrated in larger tumours and the number of vessels with LVI (*ρ*=0.46, *P*<0.001) and BVI (*ρ*=0.46, *P*<0.001) was correlated with the size of the tumour. The presence of LVI or BVI was not associated with the number of blocks investigated, but the number of vessels with LVI (*ρ*=0.34, *P*=0.001), not BVI (*ρ*=0.15, *P*=0.15), was correlated with the number of blocks.

### Blood vessel *vs* lymph vessel invasion

Lymph vessel invasion was more frequent than BVI. Sixty-six (69.5%) patients had LVI (eight only intratumoural, 35 only peritumoural and 23 both intra- and peritumoural) and 36 (37.9%) patients had BVI (12 only intratumoural, eight only peritumoural and 16 both intra- and peritumoural). In 28 (29.5%) resection specimens, both LVI and BVI were found and in eight and 38 resection specimens, only BVI or LVI were found, respectively. The presence of LVI was associated with the presence of BVI intratumourally (*P*=0.02), but not peritumourally (*P*=0.11).

**Figure 2:**
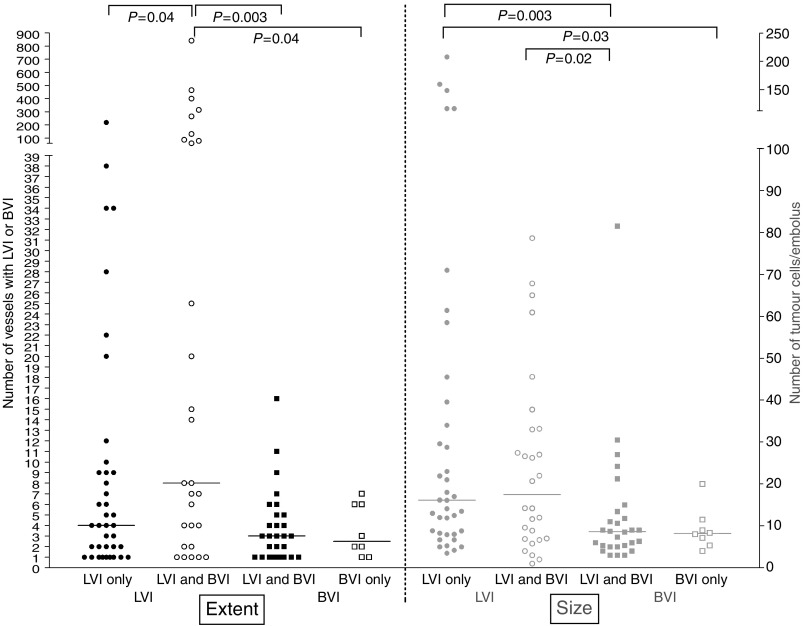
Differences in extent (left, black) and in size of tumour cell emboli (right, grey) between LVI (circles) and BVI (squares) in cases with LVI only (full circles), BVI only (open squares) or both LVI and BVI (open circles and full squares). The median value for each group is marked with a horizontal line and statistically significant differences are indicated on top (LVI=lymph vessel invasion, BVI=blood vessel invasion).

Furthermore, LVI was more extensive than BVI and lymphatic emboli were larger than blood vessel emboli. In seven cases, more than 100 vessels with LVI were found, whereas the maximum number of vessels with BVI was 16. In cases with either LVI or BVI, the median numbers of lymph and blood vessels with vascular invasion were, respectively, six (2–20.5) and three (1–5.75) (*P*=0.004) and the median sizes of the lymphatic and blood vessel emboli were 16 (8–35) and eight (5–11) intravascular tumour cells (*P*=0.001). If both LVI and BVI were present, the median of the fraction of lymph vessels of the total number of vessels with vascular invasion was 80.0% (50.0–95.4%). Figure 2 represents differences in extent and size between LVI and BVI. The median number of vessels with LVI in cases with both LVI and BVI was significantly higher than the median number of vessels with BVI in cases with both LVI and BVI (*P*=0.003) or in cases with BVI only (*P*=0.04). Furthermore, the median number of tumour cells/embolus was significantly higher in lymph vessel emboli than in blood vessel emboli, both in cases with LVI/BVI only (*P*=0.03) or in cases with both LVI or BVI (*P*=0.02).

### Immmunohistochemistry *vs* HE

On HE-stained sections it is impossible to differentiate between blood and lymph vessels. Therefore, the presence of ‘vascular’ invasion, including both BVI and LVI, was assessed. When only the results of this assessment were taken into account, 54 (56.8%) patients had vascular invasion (five only intratumoural, 38 only peritumoural and 11 both intra- and peritumoural). Both intra- and peritumourally, vascular invasion assessed on HE was associated with LVI (*p*_intra_=0.03, *p*_peri_<0.001) and with BVI (*p*_intra_=0.01, *p*_peri_=0.008). Nevertheless, the presence of LVI and BVI was missed intratumourally in 71.0 and 67.9% of immunohistochemically positive cases, respectively. Peritumourally, only 22.4 and 25% of cases with, respectively, LVI and BVI were missed on HE. Furthermore, not only the presence of LVI and BVI was underestimated on HE, but also the extent. On HE, vessels with BVI were more frequently missed than vessels with LVI: the median percentage of vessels with BVI and LVI seen on HE was, respectively, 0.0 (0.0–31.3) and 26.8 (0.0–54.0) (*P*=0.009). Overdiagnosis of ‘vascular’ invasion on one or more HE-stained slides was seen in 23 (24.2%) patients.

### Correlation to LN metastases and other clinico-pathological variables

The presence of peritumoural (*P*=0.01) ‘vascular’ invasion on HE was associated with the presence of LN metastases. The presence of peritumoural LVI was strongly associated with the presence of LN metastases (*P*=0.002). For intratumoural LVI, a trend to a positive association was found (*P*=0.07). Neither the presence of intra-, nor the presence of peritumoural BVI was associated with LN involvement (Table 3). The negative predictive value for intratumoural ‘vascular’ invasion, peritumoural ‘vascular’invasion, intratumoural LVI, peritumoural LVI, intratumoural BVI and peritumoural BVI were 60.3, 68.0, 61.9, 75.0, 57.6 and 60%, respectively. The positive predictive values were, respectively, 68.8, 57.1, 58.1, 56.5, 58.1, 50.0 and 58.3%. Multivariate logistic regression analysis revealed that peritumoural LVI (*β*=1.38, *P*=0.003) was the most important determinant of axillary involvement. Table 4 represents the associations between the presence of LVI and BVI and other clinico-pathological variables.

## DISCUSSION

We used the combination of the lymph endothelium-specific marker D2-40 and the panendothelial marker CD34 to detect and distinguish between LVI and BVI. LVI and BVI were found in 69.5 and 37.9% of patients, respectively. Other authors report LVI and especially BVI in BC to be less frequent: prevalences of LVI and BVI range from 8.8 (Nime *et al*, 1977) to 86% (Kahn and Marks, 2002) and from 4.2 (Lauria *et al*, 1995) to 33% (Kato *et al*, 2002), respectively. The presence and extent of LVI and BVI correlated with the size of the tumour and only the extent of LVI correlated with the number of blocks investigated. Therefore, the high number of FFPE tissue blocks that was investigated per resection specimen cannot fully explain the increased frequency of LVI and BVI in this study. On the contrary, the use of IHC with endothelial markers to detect LVI and BVI accounts for their high prevalence. Immunohistochemistry is a very sensitive and specific technique. In our study, the presence of LVI or BVI was missed on HE in 71.0 and 67.9% of specimens intratumourally and in 20–25% of specimens peritumourally. Furthermore, also the extent of LVI and BVI was underestimated on HE. Differences in endothelial markers and in study populations further lead to differences in LVI and BVI prevalence. For LVI, the highest prevalences are found in the studies with D2-40 and podoplanin. Kahn *et al* demonstrated lymphatic invasion in 44% of LN negative and 86% of LN positive (overall 66%) BC patients (Kahn and Marks, 2002). Recently, it has been shown that the D2-40 antibody specifically recognises podoplanin (Schacht *et al*, 2005) and that both antibodies against podoplanin and D2-40 can be regarded as reference standards for the identification of lymphatic vessels in most settings. Both anti-podoplanin and D2-40 antibodies have a sensitivity and specificity for lymphatic endothelium of over 95% (Evangelou *et al*, 2005). We previously showed that in BC, D2-40 is the best marker for lymphatic endothelium (Van der Auwera *et al*, 2005). Most studies investigating BVI in BC used FVIII or a combination of FVIII and van Gieson elastica staining. Nevertheless, CD34 or CD31 is prefered to visualise blood vessels in solid tumours (Vermeulen *et al*, 1996, 2002), as FVIII expression is absent in some tumour capillaries that can lead to underestimation of the presence and extent of BVI (Vermeulen *et al*, 1995). As CD34 is not very specific for blood vessels, especially in tumour tissue, the combination with a specific lymphatic endothelium marker such as D2-40 is necessary to differentiate between LVI and BVI. In the present study, more than 50% of lymph vessels did show CD34 expression (data not shown). CD31 might be a more specific endothelial marker than CD34, nevertheless the latter was prefered in this study, as CD31 positivity of inflammatory cells might hamper interpretation.

Recently, peritumoural lymphovascular invasion has been included in the St Gallen guidelines for adjuvant therapy of operable breast carcinoma patients (Goldhirsch *et al*, 2005). In these criteria, peritumoural lymphovascular invasion is assessed on HE-stained sections during routine pathological examination, making it impossible to differentiate between LVI and BVI. In our data set, peritumoural ‘vascular’ invasion on HE and peritumoural LVI, not BVI were correlated with the presence of LN metastasis, still the only most important prognostic factor in BC. Multivariate analysis showed peritumoural LVI to be the most important determinant for the presence of LN metastases. Together with the increase in negative predictive value to 75% for peritumoural LVI, our results suggest that immunohistochemical detection of lymphovascular invasion and differentiation between LVI and BVI might be of value in clinical practice. Further studies are needed to address this issue.

If LVI and BVI are the morphological correlates of BC cells metastasising, respectively, via the lymphatic and the haematogenous route, our data support BC metastasis models stating that haematogenous and lymphatic metastasis are two complementary but specific metastasis pathways in BC. These newer models are in contrast with older models explaining BC metastasis as a stepwise cascade going from primary tumour via regional LNs to distant sites (Pantel and Brakenhoff, 2004). Only peritumoural LVI, and not BVI, was associated with the presence of LN metastases. Other authors found a correlation between BVI and LN metastases (Lauria *et al*, 1995; Kato *et al*, 2003). This might be due to the fact that the methods they used were too insensitive to discriminate between LVI and BVI, leading to misinterpretation of BVI as LVI. Another factor contributing to the correlation between BVI and LN status in some studies might be the association between LVI and BVI. In our study, the presence of LVI was associated with the presence of BVI intratumourally, not peritumourally. As BVI is correlated with angiogenesis (Kato *et al*, 1999, 2001, 2003) and LVI is correlated with lymphangiogenesis (Schoppmann *et al*, 2004), the association between LVI and BVI might be due to the cross-interaction between lymphangiogenesis and hemangiogenesis. The hemangiogenic factors vascular endothelial growth factor (VEGF)-A, basic fibroblast growth factor, angiopoietin-1 and -2 and platelet-derived growth factor have been shown to induce lymphangiogenesis and the lymphangiogenic factor VEGF-C can also induce hemangiogenesis (reviewed in Cao, 2005; Tammela *et al*, 2005). In a study of 29 invasive breast carcinomas, Choi *et al* (2005) reported a correlation between blood and lymph vessel microvessel density. The presence of a fibrotic focus is a surrogate marker for hypoxia-driven angiogenesis (Colpaert *et al*, 2003a) and for lymphangiogenesis in BC (Van der Auwera *et al*, 2005). In the present study, the presence of a fibrotic focus was indeed correlated with the presence of both LVI and BVI. The hypothesis that blood and lymph vessels are not just different routes that cancer cells can use to metastasise, but are characterised by a different biology is furthermore sustained by the fact that some patients exclusively show BVI or LVI and by the differences in size and number between LVI and BVI. In BVI, less vessels are involved and the size of the tumour emboli is smaller than in LVI. Very extensive vascular invasion is not found in BVI. To what extent these differences influence the metastatic capacity of both pathways remains to be elucidated.

In conclusion, we demonstrated that the described immunohistochemical technique made it possible to discriminate between BVI and LVI in BC and enabled a more sensitive detection of LVI and BVI and a better assessment of the extent of both than on conventional HE stains. Furthermore, our data demonstrate that most (lympho)vascular invasion in BC is LVI and that lymph vessel tumour emboli are larger than blood vessel tumour emboli. This suggests that LVI and BVI are not just different routes of BC metastasis, but that both pathways are characterised by a different biology.

## Figures and Tables

**Table 1:** Clinico-pathological data of patients included

***N*=95**	**Clinico-pathological data**
Mean age (years)	60.5 (33.5–86.1)
*T status*	
T1	63
T2	27
T3	3
T4	2
	
*N status*	
N0	52
N1	27
N2	10
N3	5
	
*Histological type*	
Ductal	72
Lobular	10
Special type	13
	
*Tumour grade*	
I	31
II	35
III	29
	
*ER status*	
Negative	25
Positive	70
	
*PR status*	
Negative	32
Positive	63
	
*p53 status*	
Negative	73
Positive	22
	
*HER2/neu status*	
Negative	91
Positive	4
	
*Fibrotic focus*	
No	56
<1/3rd tumour diameter	20
>1/3rd tumour diameter	19
	
*Growth pattern*	
Infiltrative	15
Mixed	68
Expansive	12

ER=oestrogen receptor; N=nodal; PR=progesterone receptor; T=tumoural.

**Table 2:** Decision table for the assessment of LVI or BVI based on the CD34/D2-40 staining profile of the vessel wall

	**D2-40−**	**D2-40+**
CD34−	*‘Overdiagnosis’ on HE*	Lymph vessel *‘Picked up’ or ‘missed’ on HE*
CD34 +	Blood vessel *‘Picked up’ or ‘missed’ on HE*	

BVI=blood vessel invasion; HE=haematoxylin–eosin; LVI=lymph vessel invasion.

**Table 3:** Cross-tabs showing the association between LVI (upper part) or BVI (lower part) and LN involvement

	**LN involvement**	
	**No**	**Yes**
*‘vascular’invasion (assessed on HE)*		
Intratumoural (*P*=0.03)		
No	47	31
Yes	5	11
Peritumoural (*P*=0.01)		
No	31	14
Yes	21	28
		
*LVI (assessed on IHC)*		
Intratumoural (*P*=0.07)		
No	39	24
Yes	13	18
Peritumoural (*P*=0.002)		
No	27	9
Yes	25	33
		
*BVI (assessed on IHC)*		
Intratumoural (*P*=0.50)		
No	38	28
Yes	14	14
Peritumoural (*P*=0.12)		
No	42	28
Yes	10	14

BVI=blood vessel invasion; HE=haematoxylin–eosin; IHC=immunolistochemistry; LN=lymph node; LVI=lymph vessel invasion.

**Table 4:** Correlation between clinico-pathological variables and LVI and BVI

	**LVI**			**BVI**		
	**Pos**	**Neg**		**Pos**	**Neg**	
*T status*						
T1	39	24	P^*^=0.03	17	46	P^*^=0.001
T2	23	4		17	10	
T3	2	1		1	2	
T4	2	0		1	1	
						
*N status*						
N0	33	19	*P*=0.05	16	36	*P*=0.14
N1	18	9		11	16	
N2	10	0		5	5	
N3	5	0		4	1	
						
*Histological type*						
IDA	55	17	*P*=0.009	32	40	*P*=0.07
ILA	3	7		2	8	
Special Type	8	5		2	11	
						
*Tumour grade*						
1	15	16	*P*=0.005	4	27	*P*<0.001
2	26	9		12	23	
3	25	4		20	9	
						
*ER*						
Pos	47	23	*P*=0.41	25	45	*P*=0.46
Neg	19	6		11	14	
						
*PR*						
Pos	42	21	*P*=0.41	21	42	*P*=0.20
Neg	24	8		15	17	
						
*p53*						
Pos	18	4	*P*=0.15	10	12	*P*=0.40
Neg	48	25		26	47	
						
*HER2/neu*						
Pos	3	1	*P*=1.00	3	1	*P*=0.15
Neg	63	28		33	58	
						
*Fibrotic focus*						
Absent	33	23	*P*=0.02	15	41	*P*=0.005
<1/3th tumour diameter	18	2		8	12	
>1/3th tumour diameter	15	4		13	6	
						
*Growth pattern*						
Infiltrative	9	6	*P*=0.66	2	13	*P*=0.09
Mixed	48	20		28	40	
Expansive	9	3		6	6	

BVI=blood vessel invasion; ER=oestrogen receptor; LVI=lymph vessel invasion; N=nodal; Neg=negative; Pos=positive; PR=progesterone receptor; T=tumoural.

P^*^: *P*-value of a 2 × 2 table only including T1 and T2 tumours.
